# Enhancing Spectral Efficiency of 6G Downlink Beamforming via Cooperative Multi-Agent Deep Reinforcement Learning

**DOI:** 10.3390/s26030950

**Published:** 2026-02-02

**Authors:** Ali Al Janaby, Hussain Al-Rizzo, Yahya Qassim

**Affiliations:** 1 Department of Communications Engineering, College of Electronics, Nineveh University, Mosul 41002, Iraq; 2 Yupo Chan School of Engineering and Engineering Technology, Donaghey College of Science, Technology, Engineering, and Mathematics, University of Arkansas Little Rock, Little Rock, AR 72204, USA; hmalrizzo@ualr.edu; 3 Medical Devices Engineering Department, Engineering Technical College, Alnoor University, Mosul 41002, Iraq; yahya.taher@alnoor.edu.iq

**Keywords:** beamforming, downlink, 5G systems, reinforced learning

## Abstract

This paper presents a new beamforming algorithm for Multi-User Multiple-Input Multiple-Output (MU-MIMO) systems using Multi-Agent Reinforcement Learning (MARL). The proposed approach is shown to significantly enhance the efficiency and performance of future wireless communication systems. The system comprises two base stations, each equipped with a Uniform Rectangular Array (URA) of directional antennas. Each base station has RL algorithms that use beamforming to provide the optimal Signal-to-Interference-Plus-Noise Ratio (SINR) for each user. These algorithms also work with the other base stations to prevent user interference and ensure efficient resource use. Simulation results demonstrate that the potential of the proposed method has the potential for dynamically adapting beam patterns and maintaining high SINR across the network, resulting in more than a 2-fold improvement in throughput and a 5453% improvement in SINR.

## 1. Introduction

The rapid evolution of wireless communication technologies, particularly the advent of 5G networks, necessitates efficient management of radio resources to support the increasing demand for high data rates and low latency [1]. Multi-user MIMO systems are pivotal in 5G networks because they can simultaneously serve multiple users by utilizing spatial diversity [2]. However, one of the critical challenges in such systems is optimizing beamforming to ensure each user receives the best possible signal quality while minimizing interference from other users and the Base Station (BS) [3]. Traditional beamforming techniques struggle to adapt to the dynamic nature of wireless environments, where user positions and channel conditions are changing [4]. Recent advancements in wireless communication have led to the development of 5G technology, which has significantly improved upon previous generations by offering higher data rates, lower latency, and support for advanced applications such as virtual and augmented reality [5]. However, despite these advancements, 5G technology faces challenges in meeting the growing demands for data traffic and the increasing number of connected devices, prompting the development of 5G6G technology [6]. Research into 5G6G aims to address 5G’s limitations of 5G by introducing novel technologies and methodologies. 5G6G is expected to integrate advanced technologies such as artificial intelligence, machine learning, and block chain, pushing the boundaries of wireless communication further [7]. The vision for 5G6G includes a fully integrated, intelligent network capable of supporting new applications such as holographic communication and high-fidelity mobile holograms, with a focus on dramatically improving data rates, latency, and overall network efficiency [8].

This paper presents a beamforming solution based on MARL. Each base station in the network operates its RL agent to optimize beamforming for the users it serves. Decentralized RL agents coordinate via shared network parameters and coverage-based checks to prevent overlapping user assignments and ensure efficient resource utilization. The proposed approach dynamically adjusts beamforming weights to maximize each user’s SINR.

The rest of this paper is organized as follows. Previous works related to beamforming in the context of our proposed approach are presented in Section 2. Section 3 presents the mathematical model of the proposed AI beamforming. Section 4 introduces the system assumptions and configuration of the proposed model. Section 5 presents simulation scenario results. Section 6 presents simulation results and discussion. Finally, the conclusions and future work are provided in Section 7.

## 2. Related Works

In 2021, a Deep Reinforcement Learning (DRL) approach was proposed for hybrid beamforming in multi-user millimeter-wave (mmWave) wireless systems [9]. This method focused on optimizing the hybrid beamforming matrix while accounting for the time-varying nature of wireless channels. The primary advantage of this approach is its ability to dynamically adapt the beamforming matrix, improving performance in environments with fluctuating channel conditions. However, the complexity of implementing DRL in real-time applications posed a significant challenge due to the high computational demands required for training and deployment [10]. Another notable contribution in 2021 was the development of an online sparse beamforming method for Cloud Radio Access Networks (C-RAN) [11]. This technique used a DRL framework to optimize the activation and beamforming of Remote Radio Heads (RRHs), aiming to maximize the sum rate while adhering to power constraints. The approach was effective in reducing power consumption and enhancing network efficiency. However, its ability to make real-time decisions was limited, particularly in rapidly changing network environments, leading to suboptimal performance [12]. In 2022, researchers introduced a fast MIMO beamforming technique via DRL, designed explicitly designed for high-mobility mmWave connectivity. This method aimed to minimize latency and increase throughput by rapidly adjusting beamforming patterns. While it excelled in high-mobility scenarios, ensuring robust connectivity, it was somewhat less efficient in terms of spectral and energy usage than other techniques [13]. The following year, 2023, witnessed the development of a deep reinforcement learning-based coordinated beamforming technique for mmWave massive MIMO vehicular networks. This method significantly improved spectral and energy efficiency by coordinating beamforming across multiple users. Despite these benefits, the technique required extensive training data and computational resources, which could limit its scalability and practical deployment in large networks [14]. In 2024, a study on artificial-intelligence-enhanced beamforming for power-efficient user targeting in 5G networks explored integrating AI and reinforcement learning to optimize beamforming decisions dynamically [15]. This approach offered improved power efficiency and signal integrity, particularly in dense urban environments. However, integrating AI increased computational complexity, making real-time applications challenging [16]. In 2024, the most recent advancements introduced AI-enhanced adaptive vertical beamforming techniques tailored for 5G networks. These techniques utilized reinforcement learning to optimize both horizontal and vertical beamforming, greatly enhancing signal coverage and reliability in densely populated areas. The method showed superior performance in terms of throughput and noise resilience. However, it also faced challenges related to latency and the need for precise data to achieve effective beamforming [17,18]. Recently, Zhao et al. applied generative AI to Low-Altitude Economy Networks (LAENets), using a diffusion-based Mixture of Experts (MoE) transformer actor to optimize secure beamforming under position, channel, and eavesdropper uncertainties, reporting up to 44% gains in the worst-case secrecy rate in drone-assisted scenarios. In contrast, our work targets terrestrial 5G deployments, where multiple base stations coordinate via deep Q-networks to jointly assign users and adapt continuous MVDR-based beamforming weights under Rayleigh fading and Additive White Gaussian Noise (AWGN). This multi-agent RL framework inherently balances load, prevents beam collision, and delivers end-to-end SINR and throughput improvements. In [19], a study presents a covert communication framework in which a probabilistic, uninformed jammer helps Alice avoid detection by the Warden. By analyzing how the Warden’s detection performance varies with the jammer’s transmission probability and power distribution, the authors identified optimal strategies that enhance the transmitter’s, the jammer’s, and the overall system’s covert throughput. The authors showed that, unlike previously studied continuous jamming strategies, the optimal minimum jamming power in this probabilistic approach is non-zero and often matches Alice’s transmit power. This demonstrates that probabilistic jamming surpasses continuous jamming by achieving higher covert throughput under the same covertness and power constraints. Finally, in [20] the authors explored the limits of clandestine communication when overt users activate at random, showing that user confusion can outperform traditional power-hiding methods in single- and multi-frame scenarios.

It should be noted that existing works address parts of 6G beamforming, including DRL for adaptation, mssulti-agent coordination, 3D beamforming, and generative AI for security. However, no prior work integrates

1. Multi-agent RL with MVDR for joint user assignment and 3D beamforming;
2. Scalable architecture suitable for dense 6G deployments;
3. Explicit joint azimuth–elevation optimization;
4. Validation showing 5G techniques that scale to 6G requirements.

Our contribution fills this gap by presenting an end-to-end framework validated in 5G-realistic scenarios (28 GHz, 625 elements, Rayleigh fading) while incorporating 6G-essential features (3D beamforming, multi-agent coordination, AI-native design).

The proposed framework is inherently scalable due to its decentralized architecture. For a network with B base stations and U users, each base station operates an independent RL agent with its own Q-network. The state space for each agent scales linearly as O(U), while the action space remains constant at O(B), regardless of network size. This is because each agent only needs to decide user assignments within its coverage area, avoiding the exponential state-space growth (O(U^B^)) that would occur in a centralized approach. The coordination mechanism scales efficiently through

1. Shared network topology: All agents access a standard graph G = (V, E) where V represents base stations and E represents coverage overlaps.
2. Distributed covariance computation: Each BS independently computes its 625 × 625 covariance matrix using only local observations.
3. Coverage-based pre-filtering: The coverage check reduces the adequate action space from B to typically 1–2 neighboring BSs per user.

Adding k new base stations requires only

- Instantiating k new identical Q-networks (no architectural redesign).
- Updating the shared topology graph with k new nodes.
- No retraining of existing agents due to transfer learning properties of the reward function.

## 3. System Model

### 3.1. Multi-User MIMO System Architecture

**Figure 1:**
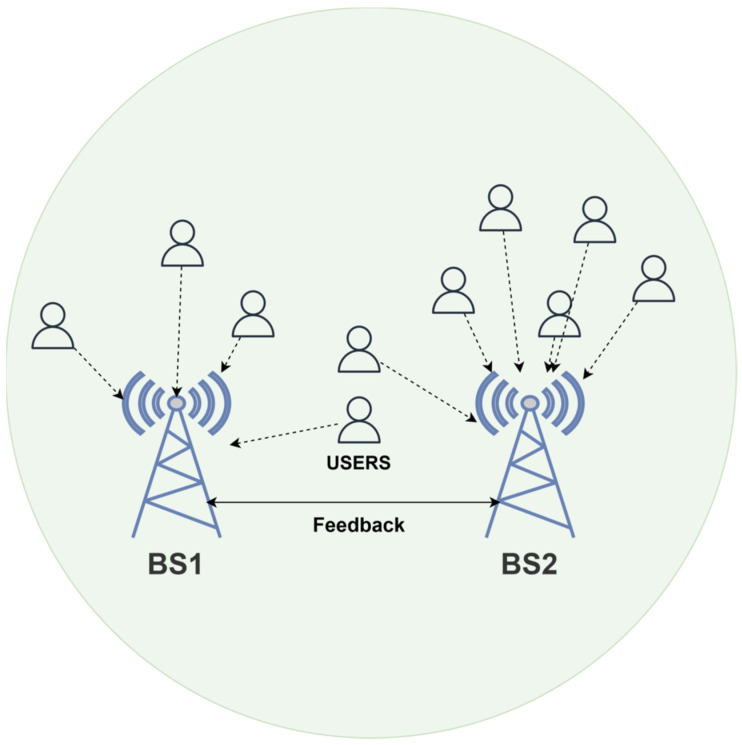
System model architecture.

As shown in Figure 1, the proposed system comprises two base stations equipped with a URA of directional antennas. The antennas are modeled using a cosine pattern to focus energy in specific directions, thereby improving signal strength for users. Users are randomly distributed across the coverage area, and each base station serves the users closest to it.

Scalability is inherent in the proposed formulation: the system is defined for a general number of base stations B and users U, where a local agent that makes decisions using only local observations and shared network parameters controls each BS. As a result, the learning and inference complexity scales approximately linearly with B, enabling deployment in dense multi-cell networks beyond the two-BS illustrative case used in our simulations.

**Figure 2:**
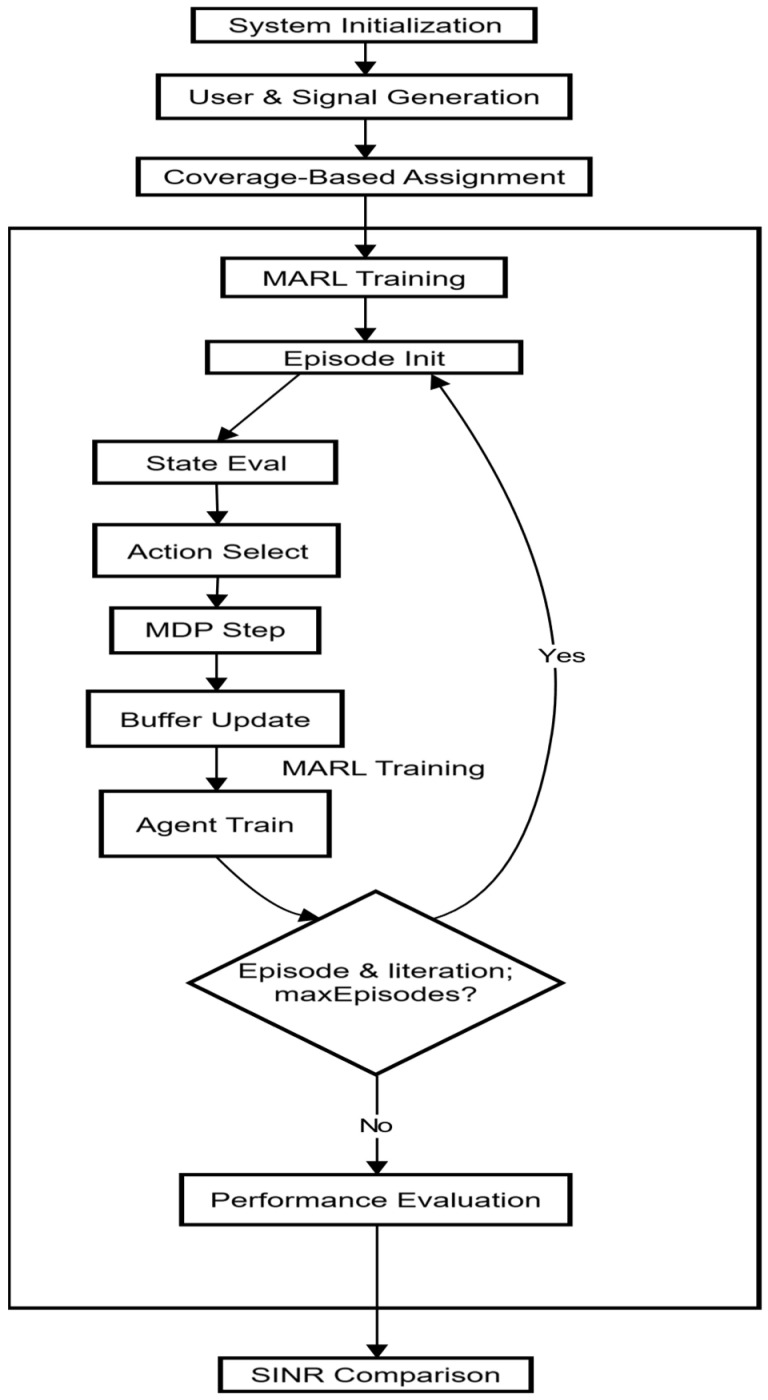
System’s flowchart.

The flowchart in Figure 2 depicts the system’s states and its operation. The flowchart illustrates the sequential process of user assignment and beamforming optimization in a multi-user system, leveraging MARL techniques.

The process begins with the initialization of system parameters, including key variables such as the number of BSs and users, antenna arrays, and reinforcement learning parameters such as the learning rate α and the discount factor. Gamma γ, Exploration rate ϵ, and the Q-table used for decision-making. Next, the system generates user signals, with each user transmitting data over a specific time window. Afterward, the BSs and users are positioned in the environment according to predefined coordinates, followed by the calculation of initial Angles of Arrival (AoA) based on the user locations relative to the BSs, which determine the direction from which each user’s signal arrives at the BS. Once the AoA of azimuth and elevation is calculated, the system enters the RL episode loop, which iterates over a set number of episodes. At the start of each episode, user assignments are initialized to reset the system’s state, ensuring that previous allocations do not affect the current episode. In the episode, the system checks each user’s assignment status. If a user is not yet assigned to a BS, the system calculates the distance between the user and all BSs to determine proximity. Based on the calculated distances, the system uses an explore-exploit decision mechanism: it either explores by assigning the user to the nearest base station or exploits the current knowledge in the Q-table to assign the user to the base station that optimizes rewards based on previous episodes. After assigning the user to a BS, the user’s wave is collected by the corresponding base station collects the user’s wave—the collected signals then pass through the Rayleigh and AWGN channels, which introduce noise and path loss. The signals from all users assigned to that base station are then combined to form the overall received signal.

To improve the quality of the received signals, the system applies Minimum Variance Distortionless Response (MVDR) beamforming [20], which focuses the array response toward the intended signal while minimizing interference from other directions. After beamforming, the SINR of the beamformed signal is calculated, and this value is used to compute a reward that guides the RL algorithm. The Q-table is updated based on the reward received, aiming to improve the system’s decision-making in subsequent episodes. As the episode progresses, epsilon (ϵ) decays, reducing the exploration rate and encouraging the system to rely more on exploitation as it learns the optimal weight assignment and beamforming strategies. This process repeats for all users in each episode until all users are successfully assigned to base stations. If there are additional episodes to run, the system proceeds to the next episode, iteratively refining then iterates to refine the Q-table further. The system algorithm is shown below (Algorithm 1):
**Algorithm 1** SDWAN-based UE–BS Assignment & Q-learning1.  **for** BS ← 1 to $N_{BS}$ **do**2.    arrays [BS] ← URA (spacing = λ/2)3.    BS pos [BS] ← e.g., (0, 0, 0), (100, 0, 0)4.  **end for**5.  **for** you ← 1 to $N_{UE}$ **do**6.    UEpos[u] ← (rand × 100, rand × 50, 0)7.  **end for**8.  Generate signals $x_{UE}[u]$ with short pulses9.  **for** you ← 1 to $N_{UE}$ **do**10.  $d_{bs}\;\leftarrow \;∥UEpos[u]\;-\;BS\;pos[bs]\;∥\;\forall \;bs$11.  **if** $min(d_{bs})\;\leq \;R_{cov}$ **then**12.     $coverage[u]\;\leftarrow \;arg\;{min}_{bs}\;d_{bs}$13.  **else**14.     coverage[u] ← 015.  **end if**16.**end for**17.**for** you ← 1 to $N_{UE}$ **do**18.   ▷ (Optional placeholder for extra per UE processing)19.**end for**20.**MDP**: $\left(s,\;a,\;r\right)\to \;Q_{BS}$21.**for** episode = 1 to max EP **do**22.    **for** iteration = 1 to numIter **do**23.       $\pi \;\leftarrow \;shuffle\{1,\ldots ,\;N_{UE}\}$24.       **for** u ∈ π **do**25.         $coverage[u]\;\leftarrow \;arg\;{min}_{bs}\;\;d_{bs}$26.         ${bs}^{*}\;\leftarrow \;arg\;{max}_{bs}\;Q_{bs}(s)$27.         $\left(s_{next},\;r)\;\leftarrow \;envStep(u,\;bs^{*},\ldots \right)$28.         $replayUpdate(Q_{bs^{*}}\;)\;\leftarrow \;(s,\;bs^{*},\;r,\;s_{next})$29.       **end for**30.    **end for**31.    **if** $\epsilon \;<\;max(\epsilon_{decay},\;\epsilon_{min})$ **then**32.       ▷ (Epsilon update condition)33.    **end if**34.**end for**35.**return** S INR, coverage, final Q-nets, etc.

### 3.2. Beamforming Model

Each BS employs adaptive beamforming using the MVDR technique. The MVDR beamformer is designed to minimize interference from other directions while maintaining the signal from the desired direction. The covariance matrix of the received signals is estimated from 2000 samples collected at the base station, and the beamforming weights are calculated to maximize each user’s SINR.

### 3.3. Reinforcement Learning Applied to Beamforming

Each BS operates an independent RL agent to optimize its beamforming. The discretized beamforming weights define the state space, and the action space represents adjustments to these weights. The reward function includes an SINR-based term and a penalty of −2 per user assigned to the same BS to discourage overlapping assignments, allowing the RL agent to maximize SINR. To prevent interference.

1. RL-Based Beamforming at BSs

The discretized beamforming weights define the state space, and the action space represents adjustments to these weights. The reward function is based on each user’s SINR, encouraging the RL agent to maximize it. The problem is formulated as a Markov Decision Process (MDP), where the state represents the user being served, the action corresponds to assigning the user to a BS, and the reward is based on the SINR achieved after beamforming. The Q-learning algorithm updates the Q-table iteratively, enabling the RL agent to learn optimal weights through experience.

2. State, Action, and Reward Definitions

The state space in the RL framework represents the users who need to be assigned to a BS. If there are (N) users, the state space consists of all possible users, indexed from 1 to (N). Each state corresponds to the RL agent that evaluates a specific user for assignment.

The action space represents the specific decisions the agent can take, namely, which BS should serve the current user. In this two-base-station scenario, each user’s action space consists of two choices: assigning the user to BS 1 or BS 2.

In addition to user–BS association, each agent also selects a 3D beamforming action defined by a joint azimuth–elevation index ($a_{az},a_{el}$) drawn from a discrete codebook. The corresponding codebook weight is constructed $w_{cb}\left(a_{az},a_{el}\right)=normalize\left(w_{aa}\left[a_{az}\right]⊙w_{el}\left[a_{el}\right]\right)$, where ⊙ denotes elementwise multiplication. To clearly relate the MARL policy to MVDR, we adopt a hybrid beamforming strategy in which the applied weight is $w=normalize\left(\rho \cdot w_{MVDR}+\left(1-\rho \right)\cdot w_{cb}\right)$, with ρ = 0.7. In this way, MARL jointly optimizes association and 3D steering, while MVDR provides a physically grounded baseline and initialization.

In Equation (1), we define the assignment reward. The hyperbolic tangent of a weighted sum of four terms, each of which captures a different aspect of user-to-base station assignment:

(1) $$r=\tanh(w_{SINR}∆SINR+w_{d}\left(1-\frac{d}{R_{c}}\right)+w_{e}\cos\left(\theta_{el}\right)-w_{l}\frac{L}{U})$$

p$R\;=\;SINR_{current}\;-\;SINR_{baseline}$, measures the gain in signal quality when user u is assigned to its chosen base station. The distance d between the user and that base station is normalized by the coverage radius, $R_{c}$, so that users closer to the BS receive a higher proximity bonus. $\theta_{el}$ is the elevation angle. Finally, L is the current load, which is the number of users served at that station, and U is the total number of users. So L/U penalizes U, P penalizes overload. We choose $w_{SINR}\;=\;0.5$ to place primary emphasis on SINR improvements, $w_{d}\;=\;0.3$ to moderately reward proximity, and $w_{e}\;=\;0.2$, so that each has fair use of elevation and load balancing are used fairly. The outer tanh bounds between −1 and +1, ensuring stable learning updates.

Equation (2) specifies the beamforming reward. $r_{bf}$, which depends only on the change in SINR produced by a candidate weight adjustment:

(2) $$r_{bf}=\{\begin{array}{c} 1-\exp\left(-\frac{∆SINR}{\alpha }\right),\;\;\;\;\;\;\;\;\;\;\;\;\;\;\;\;\;\;\;\;\;\;\;\;\;\;\;\;\;\;∆SINR>0\\ -\gamma \;\left(1-\exp\left(\frac{∆SINR}{\alpha }\right)\right),\;\;\;\;\;\;\;\;\;\;\;\;\;\;\;\;\;\;\;\;\;\;\;\;\;∆SINR\leq 0 \end{array}$$

where α = 2 controls the curvature of the exponential so that positive SINR gains yield diminishing but rewarding results, and γ = 0.7 is a negative branch, so that penalties for SINR degradations are 70% as severe as equivalent rewards; by choosing γ < 1, we make negative updates smaller in magnitude than positive ones. This prevents the agent from being discouraged by minor degradations, allowing it to continue exploring beamforming settings without a significant setback, encouraging cautious exploration without over-punishing the agent for minor missteps [21,22].

3. Q-Learning for Beamforming Optimization

The Deep Q-Network (DQN) algorithm updates the Q-values using a neural network to approximate the Q-function. Experience replay buffers and prioritized sampling enhance training stability by breaking temporal correlations and prioritizing essential transitions. The Q-value (Q(s, a)) presents the expected cumulative reward for taking action (a) in state (s) and following the optimal weights policy thereafter. The Q-values are updated using the following rule:

(3) $$Q\left(s,a\right)\leftarrow Q\left(s,a\right)+\alpha \left[r+\gamma \underset{a^{\prime }}{\max}Q\left(s^{\prime },a^{\prime }\right)-Q(s,a)\right]$$

where (α) is the learning rate, controlling how much the new information overrides the old, (γ) is the discount factor, determining the importance of future rewards, (s′) is the next state, representing the following user to be assigned, and $\left(max_{a^{\prime }}\;(Q(s,\;a)\right)$ is the maximum Q-value for the next state, indicating the best possible reward achievable from it. This update rule ensures that the RL agent gradually learns to assign users to the optimal base station and apply the appropriate beamforming configuration to minimize the interference and noise [23,24].

4. Exploration and Exploitation Strategy

The RL agent follows an epsilon-greedy policy to balance exploration and exploitation. In the early stages of learning, the agent explores different user assignments by randomly selecting actions with a probability (ϵ). As training progresses, the agent shifts toward exploitation, choosing the action with the highest Q-value more frequently. The exploration rate (ϵ) decays over time according to the following schedule:

(4) $$\epsilon =\max\left(\epsilon .decay,\;\epsilon_{min}\right)$$

where the decay factor is set to 0.995, and the minimum exploration rate ($\epsilon_{min}$) is 0.01. This decay schedule ensures that the agent continues to explore new strategies early in the training process but focuses on exploiting the learned policy as training progresses [25].

5. Coordination between Base Stations

To prevent interference between BSs, a coordination mechanism ensures that each user is served by only one base station at a time. The RL agents decide which base station to assign to the user to avoid cross-interference. The decision is based on the SINR achieved for the user at each base station. The BS with the highest achievable SINR is selected to serve the user, thereby maximizing communication quality while minimizing interference.

6. RL Agent Training and Convergence

The RL agent at each BS is trained over 200 episodes, each containing 150 iterations. During each iteration, the RL agent assigns users to base stations, calculates beamforming weights, and updates the Q-values based on the rewards received. The training process continues until the Q-values converge, indicating that the agent has learned weights for user assignment and beamforming.

The convergence rate of the RL algorithm is monitored to ensure an efficient training process. In this simulation, the Q-values stabilize after approximately 150 episodes, indicating that the RL agent has successfully learned an optimal policy.

## 4. Simulation Setup

### 4.1. Simulation Environment

The simulation environment models a 5G network with two base stations and ten stationary users randomly distributed across the coverage area. Each base station is equipped with 625 antenna elements arranged in a URA. The system operates at a carrier frequency of 28 GHz, with users positioned on the ground plane. The RL training involves 200 episodes, each comprising 150 iterations.

### 4.2. Performance Metrics

The SINR attained by each user in this study serves as the primary performance metric. The system’s ability to avoid user interference and transmission delay obtained from the achievable rate implied by the measured SINR.

Latency model:

(5) $$T_{k}=T_{p}roc+d_{k}/c+L/\left(B*log2\left(1+\gamma_{k}\right)\right)$$

where $\gamma_{k}=10^{\left(SINR_{k},dB/10\right)}$.

Here, $T_{p}roc$ is a fixed processing delay, $d_{k}$ is the 3D distanceefficiently manage handoffs between user k and its serving BS, c is the speed of light, L is the packet size in bits, and B is the system bandwidth. The reported latency curves summarize the mean and 95th-percentile latency (P95) across served users for both the baseline MVDR assignment and the proposed MARL-based 3D beamforming is also evaluated.

### 4.3. Simulation Parameters

Table 1 depicts the simulation setting and parameters used in our simulation environment. The carrier frequency is chosen within the 5G mmWave spectrum to simulate realistic high-frequency communication scenarios. Wavelength determines the spacing between the elements in the antenna array and the beamforming parameters. The simulation involves two base stations to evaluate inter-base-station coordination and user assignment strategies, with ten users randomly distributed within the coverage area, representing a typical multi-user scenario in 5G networks. An extensive array is used to simulate the high beamforming capabilities of modern base stations, with directional elements spaced at half a wavelength to focus energy in specific directions. The choice of a cosine power factor of [2, 2] in the antenna radiation pattern equation is specific to the desired characteristics of the antenna’s directional radiation pattern. The cosine antenna element with a cosine power factor of [2, 2] is a directional antenna pattern where the radiated power follows a cosine function raised to a specified power. The cosine power factor indicates how sharply the antenna’s radiation pattern falls off from its maximum direction. For a cosine antenna element with a cosine power factor of [2, 2], the radiation pattern equation can be written as

(6) $$G\left(\theta ,\emptyset \right)=\cos^{2}\left(\theta \right).\cos^{2}\left(\emptyset \right)\;$$

Where G (θ, ϕ) is the normalized gain of the antenna element in the direction specified by angles θ and ϕ, θ is the elevation angle from the boresight, measured from 0° to 90°, and ϕ is the azimuth angle around the boresight, ranging from −180° to 180°.

The power factor in a cosine antenna pattern equation determines the beamwidth and shape of the main lobe. A power factor of [2, 2] means the radiation pattern falls off as cos^2^(θ) in the elevation plane and cos^2^(ϕ) in the azimuth plane. Using a power of 2 creates a pattern in which the beam is narrower and more focused than with a lower power (e.g., [1, 1]). The [2, 2] power factor is a choice made in practical antenna designs because it offers a reasonable compromise between directionality and sidelobe levels [26]. It is often used for antennas in scenarios requiring moderate directivity is required, such as in phased-array systems and beamforming applications. This makes it versatile for applications where focusing energy in a particular direction while maintaining a functional coverage area is essential. The number of samples used in signal processing and beamforming ensures better covariance matrix estimation for the MVDR algorithm. The accuracy of covariance matrix estimation improves with the number of samples. A larger number of samples provides a more reliable estimate by reducing the variance in the estimation process. When only a few samples are used, the estimated covariance matrix may not reflect the proper signal and noise characteristics, leading to inaccurate beamforming weights. If too few samples are used (e.g., significantly less than 2000), the covariance matrix may be poorly estimated, leading to erroneous beamforming. This can result in a beam that does not focus correctly in the intended direction or in increased sidelobe levels, thereby reducing the SINR and degrading performance. The AWGN N_0_ parameter is introduced to simulate real-world conditions in which the signal is corrupted by background noise, making SINR a critical performance metric. Additionally, the signals pass through the Rayleigh channel, which introduces fading, and path loss is also considered. The beamforming weights determine the direction towards the user and focus the antenna array’s radiation pattern, which the RL algorithm optimizes to maximize SINR. The beamforming weights are dynamically optimized via reinforcement learning, continuously adapting to maximize each user’s SINR. The positions of Base stations and users are on a 2D (x-y) plane with z = 0, simulating a ground-level deployment in a typical urban or suburban scenario. The base stations are placed at fixed coordinates. BS 1 is positioned at the origin (0, 0, 0), which is a common choice for simplicity. This represents a reference point in the coverage area, and BS 2 is positioned at (100, 0, 0), which places it 100 m away from BS 1 along the x-axis. This separation is intended to create a scenario in which both BSs have overlapping coverage areas but are far enough apart to require careful coordination to avoid interference and optimize user assignment. These performance metrics and simulation parameters are designed to comprehensively evaluate the proposed RL-based beamforming system, ensuring it meets the 5G network requirements for signal quality and interference management.

## 5. System Mathematical Model

We consider a wireless communication system with K users, each equipped with an isotropic antenna, and ith base station, each equipped with a URA of M antenna elements. The signal transmitted by the k-th user can be represented as

(7) $$s_{k}\left(t\right)=A_{k}x_{k}(t)$$

where $\left(x_{k}(t)\right)$ is the baseband signal transmitted by the *k*-th user, and *A_k_* is the amplitude scaling factor that accounts for the user’s transmit power [27]. The signal received by the N-element array of the ith base station at the discrete time index is collected and is expressed as

(8) $$y_{i}\left[n\right]=\sum_{k=1}^{U}\sqrt{P_{k}}{\;\;g}_{ik}\left[n\right]a_{ik}s_{k}\left[n\right]+n_{i}[n]$$

where *U* denotes the total number of users, $P_{k}$ is the transmit power allocated to user *k*, and $gik[n]\;=\sqrt{L_{ik}}\;h_{ik}[n]$ represents the composite channel gain consisting of the path loss $L_{ik}$ and the Rayleigh fading coefficient $h_{ik}[n]$. The vector $a_{ik}\in \;C^{N}$ is the steering response of the ith array in the direction of the user $k,\;s_{k}[n]$ is the user’s baseband symbol, and $n_{i}[n]$ denotes noise [28].

The spatial response of the $M_{y}\times M_{x}$A: The half-wavelength uniform rectangular array (URA) deployed at the ith base station is obtained by evaluating the phase delay at each element for a plane wave incident from the azimuth–elevation direction $\left(\theta_{ik},\;\varphi_{ik}\right)$. For user k, the resulting steering vector is expressed as

(9) $$a_{ik}\left(\theta_{ik},\;{\;\varphi }_{ik}\right)=\left[\exp\left(-j\pi \left(c\sin\theta_{ik}+r\sin\varphi_{ik}\right)\right)\right]\;\;\;\;\;\;\;r=0,\;\ldots ,\;M_{y}-1,\;c=0,\;\ldots ,\;M_{x}-1$$

where $c\;\in \;\{0,\ldots ,\;M_{x}-\;1\}$ and $r\;\in \;\{0,\ldots ,\;M_{y}-\;1\}$ enumerate, respectively, the column and row indices of the array, and the inter-element spacings are $d_{x}\;=\;d_{y}\;=\;\lambda /2$, and the carrier wavenumber is 2π/λ. In the present study, we set $M_{x}\;=\;M_{y}\;=\;25$; consequently, $a_{ik}\;\in \;C^{N}$ has length $N\;=\;M_{x}M_{y}=\;625$ [29].

The attenuation between the user k and base station i is modelled by the familiar distance-dependent expression:

(10) $$\beta_{ik}={\left(\frac{d_{0}}{d_{ik}}\right)}^{\eta },\;\;\;\;\;\;\;\;\;\;\;\;\;\;\;\;d_{ik}=|\left|p_{k}-q_{i}\right||$$

where $p_{k}\;=\;{\left[x_{k},\;y_{k},\;z_{k}\right]}^{T}$ and $q_{i}=\;\left[x_{B,i},\;y_{B,i},\;z_{B,}\right]$. Note the positions of the user and the base station, respectively. $d_{ik}$ is the corresponding Euclidean distance, $d_{0}=\;1\;m$ is a reference distance, and η > 2 is the path-loss exponent that accounts for the propagation environment [30]. Fading is incorporated via a Rayleigh coefficient that multiplies the deterministic path-loss factor in (8). The composite scalar channel gain appearing in the received signal model is therefore

(11) $$g_{ik}=\sqrt{\beta_{ik}}\;\;h_{ik}\left[n\right],\;\;\;\;\;\;\;\;\;\;\;\;h_{ik}[n]\sim CN(0,1)$$

Here, h sub i. k, open bracket n close bracket is a circularly symmetric complex Gaussian random variable with unit variance [31]. As a result, the effective array channel for user k snhts is given by the product of the outputs $g_{ik}\left[n\right]a_{ik}\;(\theta_{ik},\;\varphi_{ik})$. In forming both the covariance estimate and the beamforming codebook. After collecting NN time domain snapshots of the array output, the ith base station forms the sample covariance matrix:

(12) $$\hat{R_{i}}=\frac{1}{N_{s}}\;\sum_{n=1}^{N_{s}}y_{i}\left[n\right]\;y_{i}^{H}\;[n]$$

where $y_{i}[n]\;\in \;C^{N}$ represents the $N\;=\;M_{x}M_{y}\;=\;625$ element spatial snapshot at discrete time index n [27]. The outer product $y_{i}\left[n\right]\;y_{i}^{H}\;\left[n\right]$ captures the instantaneous spatial correlation of the received signal, averaging over the $N_{s}\;=\;2000$. Snapshots available in the measurement window provide an estimate of the array covariance under the channel assumption in (9).

The MVDR covariance matrix is estimated using *N_s_* = 2000 array snapshots (equal to the simulated baseband sample length), while the 2D beamscan AoA estimator uses a shorter burst of N_b = 100 snapshots to reduce overhead during angle estimation.

For an M-element array, estimating the sample covariance scales as O($N_{s}\cdot M^{2}$) and computing MVDR weights via matrix inversion scales as O($M^{3}$). With the 25 × 25 URA used in this work (M = 625) and *N_s_* = 2000 snapshots, the dominant terms are approximately $N_{s}\cdot M^{2}$ ≈ 7.8 × 10^8^ and $M^{3}$ ≈ 2.4 × 10^8^ (complex) operations per update, which motivates our hybrid design. In practical implementations, MVDR updates can be performed periodically and accelerated using Cholesky factorization or iterative solvers, whereas DQN inference requires only a few matrix multiplications per decision step.

With the covariance, the base station computes the MVDR beamformer for any user u that has been associated with the BS. Denoting the steering vector for that user by $a_{iu}=\;a_{ik}\;(\theta_{iu},\;\varphi_{iu})$. From Equation (7), the optimal weight vector is

(13) $$w_{iu}^{MVDR}=\frac{R_{i}^{-1}a_{iu}}{a_{iu}^{H}\;R_{i}^{-1}a_{iu}}$$

where the numerator $R_{i}^{-1}\;a_{iu}$ shapes the array response so that interference and noise arriving from all directions other than $\left(\theta_{iu},\;\varphi_{iu}\right)$ are suppressed in a minimum variance sense, while the denominator $a_{iu}^{H}R_{i}^{-1}\;a_{iu}$ normalizes the beam such that the composite response in the look direction is precisely unity; i.e., the constraint $w_{iu}^{MVDRH}\;a_{iu}=1$ is satisfied [32,33]. The resulting weight vector, therefore, extracts the desired signal at the combiner output. The beamformed signal is then

(14) $$\hat{S_{k}}\left(t\right)=w_{b}^{†}y_{b}(t)$$

where $\hat{S_{k}}\left(t\right)$ is the beamformed signal, $w_{b}$ is the beamforming weight vector designed to maximize the SINR for the k-th user, and $y_{b}$ is the user’s signal, and the SINR for a given user (k) at base station (b) is

(15) $$SINR_{i,k}=\frac{{\left|w_{i}^{H}\sqrt{L(d_{k,b})}\;H_{b,k}\;a_{b}\left(\theta_{k,i},\;\varphi_{k,i}\right)\right|}^{2}}{\sum_{j\neq k}{\left|w_{i}^{H}\sqrt{L\left(d_{j,i}\right)}\;H_{i,j}\;a_{b}\left(\theta_{j,i},\;\varphi_{j,i}\right)\right|}^{2}+\sigma^{2}}$$

where $SINR_{i,k}$ is the SINR for user k at base station i, the numerator represents the beamformed power of the desired signal from the k-th user, $w_{i}$ is the beamforming weight vector, $L\left(d_{k,i}\right)$ accounts for path loss with an exponent η, $H_{i,k}$ models Rayleigh fading, and $a_{b}\left(\theta_{k,i},\;\varphi_{k,i}\right)$ the steering vector is aligned with the azimuth and elevation angles. $\theta_{k,i},\;\varphi_{k,i}$ the k-th user, respectively. The denominator comprises two components: the first term captures the interference power from other users. j≠ k, incorporating their respective path loss, fading, and alignment through $\sqrt{L\left(d_{j,i}\right)}\;H_{i,j}\;a_{b}\left(\theta_{j,i}\right)$, while the second term, σ^2^, represents AWGN [33].

### 5.1. Reinforcement Learning for User Assignment

The system uses reinforcement learning to assign users to base stations. The RL agent’s goal is to maximize each user’s SINR by selecting the appropriate base station.

**State**: The state represents the current user being served.

**Action**: Action a corresponds to assigning the user to a base station.

**Reward**: The reward for serving the k-th user at the b-th base station is based on the SINR:

(16) $$r_{k,b}=\log_{2}\left(1+SINR_{k,b}\right)$$

where $SINR_{k,b}\;$ is derived from the SINR Equation (14).

Table 2 shows the simulation parameters used for learning.

The choice of RL parameters, such as the learning rate α, discount factor γ, exploration rate ϵ, number of episodes, and iterations per episode, is crucial for the successful training and performance of the RL algorithm. These parameters control how the RL agent learns from its environment and balances exploration with exploitation. The learning rate α determines how much the RL algorithm updates the Q-table with each new experience. It controls the step size during the learning process. A learning rate of 0.01 is relatively low, indicating that the algorithm updates the Q-values gradually based on new experiences. A smaller learning rate helps in creating more stable updates to the Q-table. It prevents the algorithm from making overly drastic changes in response to a single experience, which could otherwise lead to instability or divergence in learning. With α = 0.01, the algorithm converges more slowly, allowing it to learn the optimal policy over many episodes rather than overfitting to early experiences. This is particularly important in complex environments where initial policies might not be optimal. The discount factor, γ, represents the relative importance of future rewards versus immediate rewards. It determines how much the algorithm values long-term benefits over short-term gains. A discount factor of 0.99 is close to one, indicating a high emphasis on future rewards. A value of 0.99 means that the RL algorithm strongly considers long-term rewards when making decisions. This is beneficial in scenarios such as beamforming, where actions can have long-term impacts on signal quality and network performance. In complex environments, focusing too much on immediate rewards (γ closer to 0) can lead to myopic behavior, where the agent optimizes for short-term gains but fails to achieve optimal long-term performance. A higher γ encourages the agent to plan. The exploration rate ϵ controls the tradeoff between exploration and exploitation: an initial value of 1.0 means 100% exploration, a decay of 0.995 (means exponential decay),, and a minimum value of 0.01 (1%) stops exploration. Starting with ϵ = 1.0 means the agent initially explores the environment thoroughly by randomly selecting actions. This is crucial for gathering diverse experiences and avoiding initial biases. The decay factor of 0.995 gradually reduces ϵ after each episode. This balance allows the agent to shift progressively from exploration to exploitation. By decaying ϵ at this rate, the agent still explores enough to learn effectively, but increasingly exploits known information as it gains confidence in its policy, and setting a minimum ϵ = 0.01 ensures that there is always a slight chance of exploration, preventing the agent from getting stuck in local optima and ensuring continual adaptation to any changing dynamics in the environment.

The number of episodes represents the total number of complete cycles of interaction the RL agent has with the environment during learning to find the optimal policy. Two hundred episodes provide sufficient training time, allowing the agent to explore, learn, and refine its policy. With 200 episodes, the agent has ample time to explore the state space, know the optimal actions, and converge towards an optimal or near-optimal policy. This is especially important in environments where optimal beamforming requires understanding complex, long-term dependencies. While more episodes can lead to better policies, they also risk overfitting to the specific scenarios encountered during training. The iterations per episode denote the number of steps the agent takes in the environment. Each iteration involves choosing an action, receiving a reward, and updating the Q-table. One hundred fifty iterations per episode provide sufficient interaction for meaningful learning without making each episode computationally intensive. With 150 iterations, the agent can explore adequately in each episode, updating its policy based on diverse experiences. These parameters guide the RL algorithm’s learning, balancing exploration and exploitation to find the best beamforming solution. The parameters ensure the agent can adapt to the environment’s complexity, learn optimal actions, and maximize the overall signal quality and network performance. The Q-value for each state-action pair is updated as in (3).

### 5.2. User Assignment and Beamforming Execution

Based on the Q-values, the system assigns users to the base station that maximizes their SINR. Once assigned, the base station uses the calculated beamforming weights to direct its beam toward the assigned users, ensuring optimal signal reception. This process is repeated iteratively, with the RL agent continuously learning and updating its policy to improve the SINR throughout the weight optimization and the overall system performance.

1. Key Components of RL
  (a) States: State Space: the state space represents the users who need to be assigned to a base station. Each state s corresponds to a specific user currently being considered for assignment.
  (b) State Representation: If there are K users, the state space can be represented as 1, 2, …, K, where each number corresponds to a different user.
2. Actions:
  (a) Action Space: The action space represents the possible base stations to which a user can be assigned. Each action a corresponds to assigning the current user to one of the available base stations.
  (b) Action Representation: If there are B base stations, the action space can be represented as two, where each number corresponds to a different base station.
3. Q-Values Q (s, a):
  (a) Q-Value Definition: The Q-value represents the expected cumulative reward for taking action a in state s and following the optimal policy thereafter.
  (b) Q-Table: The Q-values are stored in a table called the Q-table, where each entry Q(s, a) corresponds to the expected reward for assigning user s to base station a.

### 5.3. Learning Process: Q-Learning

Q-learning is a popular RL algorithm used in general systems. Its goal is to iteratively update the Q-values in the Q-table so that they accurately represent the best possible actions to take in each state.

1. Initialization

The Q-table is initialized with zeros or random values. Each entry Q(s, a) represents the expected reward for assigning user **s** to the base station a.

2. Exploration vs. Exploitation
  - Exploration: The agent explores different actions to gather environmental information. It does this by choosing actions randomly with probability ϵ (epsilon-greedy strategy).
  - Exploitation: The agent exploits the knowledge acquired by choosing the action with the highest Q-value for the current state with probability (1 − ϵ). As the training progresses, the agent gradually shifts from exploration to exploitation by reducing ϵ, enabling it to focus on the most promising actions.
3. Q-Value Update

After taking an action a in state s, the agent observes the resulting reward r(s, a) and transitions to a new state s′. The Q-value for the state-action pair (s, a) is updated using the Q-learning update rule.

4. Policy Derivation

Over time, the Q-table converges to values representing the optimal policy. It states that, for any given state s, which includes parameters such as user location and SINR, the system selects the action a that maximizes the Q-value:

(17) $$\pi \left(s\right)=\arg\underset{a\in A}{\max}Q(s,\;a)$$

Since the Q-values are updated using a reward function based on SINR improvement, this policy ensures that the beamforming weights are chosen maximize the expected SINR.

### 5.4. Application in the System

1. User Assignment:

The RL agent assigns each user to the base station that maximizes their SINR based on the Q-values learned through interaction with the environment.

2. Beamforming:

Once a user is assigned to a base station, beamforming improves signal quality, ensuring the user experiences the best possible SINR.

The system uses reinforcement learning to intelligently assign users to the optimal base station, ensuring the best possible communication quality. The Q-learning algorithms enable the system to learn from experience and optimize user assignment based on the cumulative SINR achieved. Over time, the system improves its decision-making process and achieves higher performance. Next, a discussion will present how the system identifies users among interference, separates them, and how the RL system assigns users to BSs.

### 5.5. User Identification Among Noise

When signals from multiple users are received at a BS, the signal comprises the desired user signals, interference from other users, and noise. Identifying and separating the users involves the following steps:

1. Signal Reception:

Each base station receives a combination of signals from all users in its vicinity. The received signal $y_{b}(t)$. The base station for all users can be expressed as

(18) $$y_{b}\left(t\right)=\sum_{k=1}^{K}a_{b}\left(\theta_{k}\right)s_{k}\left(t\right)+n_{b}(t)$$

where ab $\left(\theta_{k}\right)$ is the steering vector for the k-th user at the b-th base station, $s_{k}(t)$ is the signal from the k-th user, and $n_{b}(t)$ is the noise.

2. Beamforming:

Beamforming is applied to enhance the signal from the desired user while suppressing interference from other users and noise. For each base station, a beamforming weight vector ($w_{b}$). It is calculated to focus on the signal arriving from a specific direction, shown in (13).

The beamforming process effectively enhances the signal of the desired user while minimizing the impact of other users’ signals and noise. This helps reduce interference and increase signal fidelity. After beamforming, the system must separate and identify each user’s signal. The system calculates the SINR for each beamformed signal $\left(\hat{s_{k}}(t)\right)$. It is calculated as in Equation (14).

After beamforming, a short burst of $N_{s}\;=\;100$ An array of snapshots are processed with a conventional 2-D Beam scan estimator to obtain rough azimuth and elevation angle estimates for every active user. Let $R_{x}\in \;C^{M\times M}.$ The sample covariance of the snapshot matrix $X\;=\;[x_{1},\;\ldots ,\;x_{N_{s}}\;]$, as in

(19) $$P_{B}\left(\theta ,\;\varphi \right)=a^{H}\left(\theta ,\;\varphi \right)R_{x}a\left(\theta ,\;\varphi \right)$$

where a(θ, φ) is the steering vector of the 25 × 25 URA given in (7). We perform a grid search over φ ∈ [−90°, 90°] and θ ∈ [−30°, 30°] with one-degree resolution and declare

(20) (θ^k, φ^k)=argmaxθ,φ PB(θ, φ)

As the estimated direction of arrival, substituting $\left(\theta_{ik},\;\varphi_{ik}\right)$ into the steering vector $a_{ik}(\theta_{ik},\;\varphi_{ik})$ defined in (7) and combining with the MVDR weights of (12) yields a beam that maximizes the SINR for that user. After each snapshot, the RL agent compares the achieved SINRs and assigns each user to the base station with the highest SINR. If the SINR value is negative, the RL policy re-evaluates itself, handing users over to different base stations, and recalibrates the weights to a different choice that produces a superior SINR. Each time a reassignment occurs, the serving BS re-computes its beamforming weights so that energy remains tightly focused on the current user. The RL agent continually updates the assignments to ensure optimal performance. The base station applies RL again to optimize beamforming and focus its antenna array on the assigned user, providing the best possible signal quality. The MVDR beamforming, angle calculation, RL-based base-station selection, and weight update run continuously, enabling the network to maintain high link quality.

## 6. Results and Discussion

The simulation results show that the proposed RL-based beamforming approach effectively maximizes each user’s SINR. The RL agents regulate beamforming weights based on user locations to maintain high SINR. The centralized RL agent effectively coordinates handoffs, preventing interference and optimizing resource allocation (RA) across the network.

**Figure 3:**
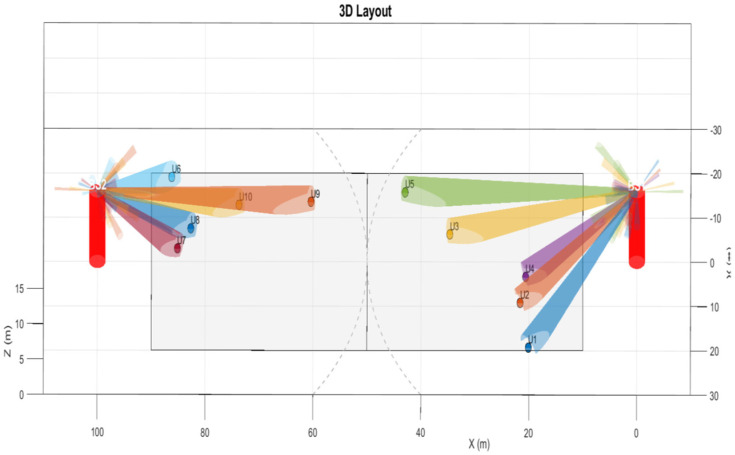
3D map showing the BSs and their individual users.

Figure 3 shows a 3D map of users and BSs, highlighting the beam directions assigned by the beamforming algorithm. BSs are shown as red circles, and users as small dots, with colored lines indicating beams directed toward each user. The beams are focused on the assigned users, indicating that the system accurately adjusts beam directions based on user location. This spatial distribution highlights the system’s ability to manage multi-user scenarios and minimize interference through appropriate beam selection and steering. These visualizations show the initial conditions before applying the MVDR beamforming technique.

**Figure 4:**
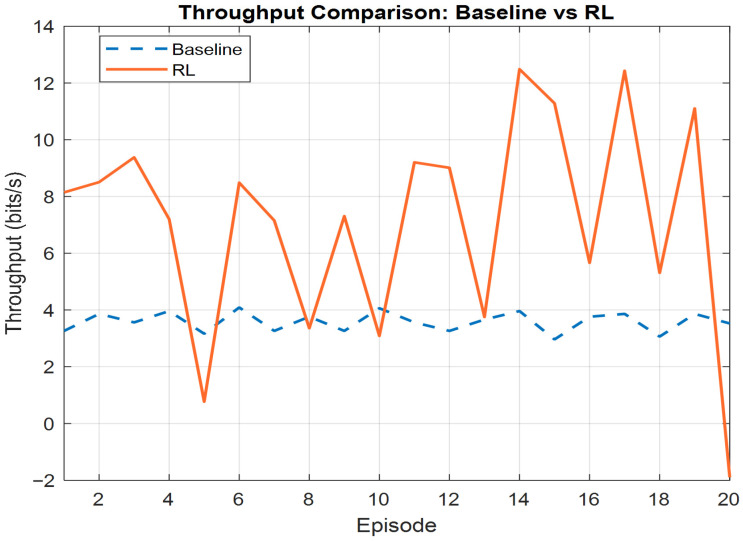
System’s throughput.

The throughput analysis in Figure 4 indicates that system throughput increases with higher SINR. Initially, without training, the system exhibits 3.4 Mbps due to lower SINR. After training and improving the SINR values for the users, the throughput increases to reach a maximum of 12 Mbps, suggesting that the system’s bandwidth capacity is fully utilized under these conditions. This demonstrates the system’s efficiency in translating improvements in signal quality into higher data rates, reinforcing the performance gains achievable through better channel conditions and beamforming.

**Figure 5:**
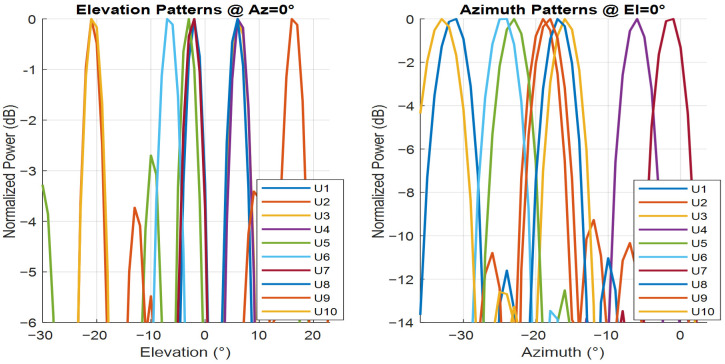
Azimuth and elevation beam power.

In the comparison of the beam patterns before and after traditional MVDR beamforming, as shown in Figure 5, the plots show the benefits of beamforming. Before beamforming, the beam pattern is directional, with energy distributed more uniformly across different directions. After beamforming, the pattern focuses the energy toward specific users. The azimuth and elevation cut provides further evidence of this, showing a more concentrated beam. This confirms that the beamforming algorithm effectively enhances signal strength and focuses energy toward users while minimizing interference in other directions.

**Figure 6:**
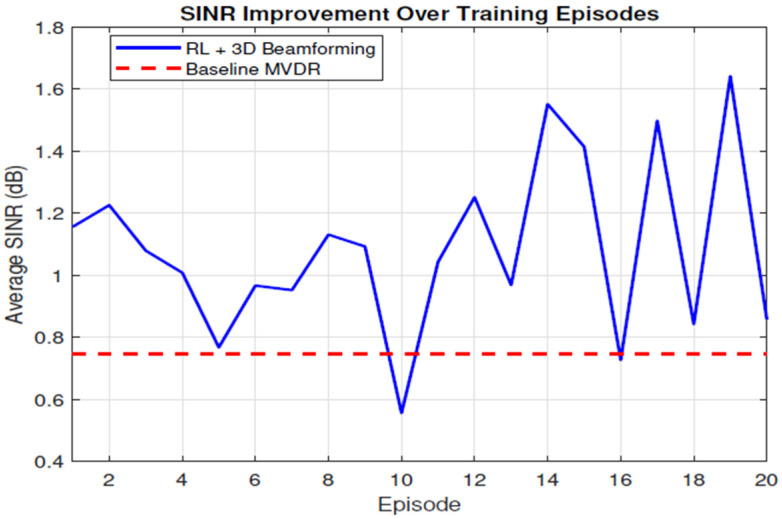
SINR improvement over time.

Figure 6 tracks the evolution of the network SINR during the episode training run. The dashed red line (0.75 dB) is the constant reference obtained with conventional MVDR weights derived from channel knowledge and deterministic steering. The solid blue line shows the performance of the proposed 3-D RL beam-steering policy. In episodes 1–5, the agent outperforms the MVDR baseline by 0.3–0.5 dB in the very first episode, but exploration occasionally drives the average down at episode 5, and this trend continues through episodes 6–12. As ϵ-greedy exploration decays, the curve climbs steadily. A stable gain of 0.30–0.55 dB over the baseline is sustained, and episode 12 reaches 1.26 dB—roughly a 0.5 dB margin, reaching episodes 13–20. Once the replay buffers are well populated, the policy refines its elevation and azimuth selections, peaking at 1.55 dB in episode 14 and 1.65 dB in episode 19, and notices that even at the lowest stage of training, the episode still matches the MVDR line. The agent converges to a mean gain of 0.6 dB over MVDR.

**Figure 7:**
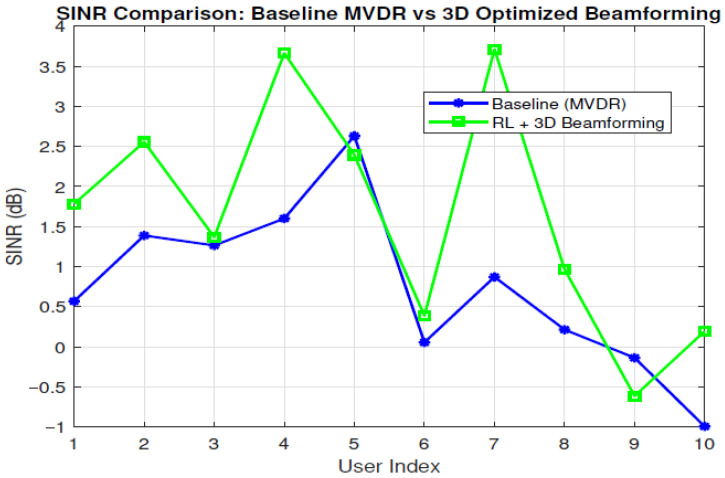
Compares the SINR values for each user.

Figure 7 plots each user index on the x-axis, and two SINR scenarios on the y-axis. We see that users experience different SINR under the RL policy compared to the baseline, showing that the RL-based assignment and beamforming strategy can reduce interference or enhance signal quality. However, certain users may occasionally show SINR values similar to or slightly lower than the baseline, reflecting the exploration-exploitation nature of RL or because the RL policy is still balancing multiple user requirements across two base stations. This could mean that the baseline MDVR has actually reached peak performance, and that when the RL tries to optimize or adjust the value, it would shift away from the optimal value chosen in the first calculation. Overall, the results underscore the potential gains in link quality from a more intelligent network compared to traditional networks.

**Figure 8:**
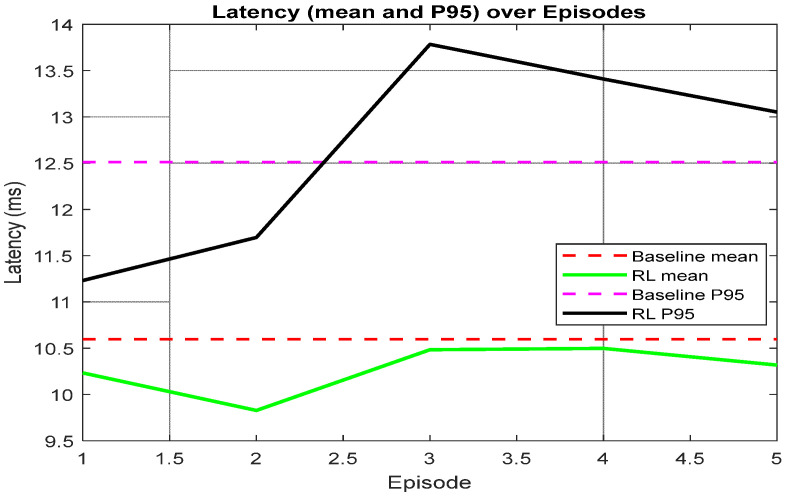
Latency (mean and P95) over training episodes.

Figure 8 depicts that the mean latency achieved by the MARL-assisted 3D beamforming remains below the baseline mean in all reported episodes, indicating that the learned assignment/beam decisions can reduce the average service delay. However, the RL P95 curve exhibits a temporary increase around Episode 3, which suggests that exploration and non-stationary policy updates can momentarily worsen tail latency for a subset of users. As training progresses, the P95 latency decreases, reflecting improved stability of the learned policy and reduced delay.

The proposed framework can be extended by incorporating a latency term into the reward to directly control tail behavior when required by URLLC-like applications.

## 7. Conclusions

This paper presents a beamforming approach for 5G networks using RL to enhance power-efficient user targeting. The proposed method influences independent RL agents at each BS to optimize channel conditions and beamforming. The system, which maximizes each user’s SINR, improves beamforming accuracy and reduces interference across the network. By integrating RL-based user assignment and MVDR beamforming, our system adapts beam patterns to the environment. This approach ensures that beamforming weights are optimized, directing energy effectively toward intended users while minimizing interference to others, thereby improving overall system efficiency. The simulation results validate our RL-enhanced beamforming strategy, which enhances communication quality in 5G networks, achieving high throughput and acceptable SINR across several scenarios. The centralized RL coordination among BSs also ensures efficient handoffs, preventing users from experiencing signal interference as they move across coverage areas. Across RL training episodes, the proposed scheme lifts the network’s mean SINR from 0.7 dB to 1.15 dB, a 54% relative increase, while the best episode peaks at 1.675 dB, and the throughput rises from 3.2 Mbps to 12 Mbps, more than doubling the data rate. This work illustrates the potential of RL to optimize beamforming in power-efficient user targeting.

Future research will extend this framework to 6G application scenarios where we will evaluate three additional scenarios: Scenario 1: high-mobility vehicular communications; Scenario 2: dense urban hotspot (6G XR Applications); and Scenario 3: multi-tier heterogeneous network. Moreover, we will expand performance metrics, beyond SINR, latency and throughput, and we will evaluate energy efficiency, fairness index (Jain’s Fairness), handoff rate.

## Figures and Tables

**Table 1:** Simulation setting and parameters.

Metric	Value
Carrier Frequency	28 GHz
Spacing among Antenna Elements	λ/2
Number of Base Stations	2
Number of Users	10
Antenna Array Configuration	25 × 25 URA (ULA with 625 elements)
Antenna Element Type	Cosine power factor
Number of Samples	2000
AWGN Power	Noise is modelled as AWGN with an SNR of 3dB
Modulation Scheme	16-QAM
Bits per Symbol	4
Symbol Rate	1 MHz
Number of Symbols per Frame	100
Eb/No Range (dB)	0–30 dB
Beamforming Weights Range	Discrete complex values, optimized via RL
Coverage Radius	50 m
Base Stations Distance Apart	100 m
Path Delay	0, 0.5 ns, 1 ns
Average Path Gains	0, −20 dB, −40 dB
Maximum Doppler Shift	50 Hz

**Table 2:** Simulation parameters.

Metric	Value
Learning Rate α	1 × 10^−4^
Discount Factor γ	0.99
Exploration Rate ϵ	Initial: 1.0, Decay: 0.995, Minimum: 0.01
Number of Episodes	200
Iterations per Episode	150

## Data Availability

The original contributions presented in this study are included in the article. Further inquiries can be directed to the corresponding author.
